# Engagement in Aerobic Exercise Is Associated with a Reduced Prevalence of Sarcopenia and Severe Sarcopenia in Italian Older Adults

**DOI:** 10.3390/jpm13040655

**Published:** 2023-04-11

**Authors:** Hélio José Coelho-Júnior, Riccardo Calvani, Anna Picca, Matteo Tosato, Francesco Landi, Emanuele Marzetti

**Affiliations:** 1 Department of Geriatrics and Orthopedics, Università Cattolica del Sacro Cuore, 00168 Rome, Italy; 2 Fondazione Policlinico Universitario “A. Gemelli” IRCCS, 00168 Rome, Italy; 3 Department of Medicine and Surgery, LUM University, 70100 Casamassima, Italy

**Keywords:** swimming, running, strength training, muscle mass, muscle strength, frailty, elderly

## Abstract

The present study was conducted to test the association between adherence to specific exercise modalities and sarcopenia severity in Italian older adults. Data were collected as part of the ongoing Longevity Check-Up 7+ (Lookup 7+) project. Lookup 7+ began in June 2015 and has since been conducted in unconventional settings (e.g., exhibitions, malls, social events) throughout Italy. In the present study, we used data on adults 65 years and older. Sarcopenia was identified according to the simultaneous presence of dynapenia and low appendicular muscle mass. Muscle strength was measured by isometric handgrip and sit-to-stand (STS) testing. Sarcopenia was categorized as severe if participants reported difficulty or inability to walk 400 m. Engagement in running and/or swimming (RS) or strength training with or without stretching (SS) was used to define exercise modalities. Analyses were conducted in 3289 participants (mean age: 72.7 ± 5.7 years; 1814 women). The results of the binary regression showed negative associations between RS and the presence of STS-based sarcopenia in women, and between RS and STS-based severe sarcopenia in men. Collectively, these findings indicate that RS is negatively associated with the presence of sarcopenia in large sample of relatively unselected Italian older adults.

## 1. Introduction

Sarcopenia is a neuromuscular disease characterized by the combination of reduced muscle strength and low muscle mass [1,2,3]. The co-occurrence of mobility deficits allows determination of sarcopenia severity [1,2,3]. According to the European Working Group on Sarcopenia in Older People 2 (EWGSOP2) [1] and the Asian Working Group for Sarcopenia (AWGS) [3], dynapenia can be identified by either isometric handgrip strength (IHG) or sit-to-stand (STS) tests. However, IHG and STS are related to different aspects of the neuromuscular function. Weak-to-moderate to high correlations have been observed between IHG and lower-limb muscle strength as well as physical performance [4,5,6,7,8]. STS is instead strongly correlated with muscle strength, power, balance, and endurance [7,9,10,11,12]. Furthermore, physical function was found to be stronger related with STS than IHG [10].

Sarcopenia is frequent in advanced age [13], with prevalence rates ranging from 10% to 27% depending on the operational definition used [13]. The condition is more prevalent in men than in women when diagnosed according to the EWGSOP2 criteria [13]. The progression of sarcopenia entails an increased risk of negative events, including malnutrition, anorexia, physical inactivity, metabolic and osteoarticular disorders, cognitive impairment, depression, falls, and death [14,15]. Therefore, the identification of lifestyle habits that might prevent the onset of sarcopenia or avoid its progression is a topic of great interest to health professionals responsible for older people’s care.

The benefits of exercise on sarcopenia have long been recognized [16] and experts in the field have suggested prescription of physical exercise as a first-line therapy to manage this condition [17]. Much attention has been paid to strength training [16], a type of exercise in which muscles contract to sustain or work against a given force [18]. Numerous studies have observed that strength training improves muscle strength, muscle mass, and physical performance in older adults with sarcopenia [16,19,20,21]. However, results were not consistent across studies [16,19,20,21]. Furthermore, most studies have examined the effects of resistance training on the defining criteria of sarcopenia, whilst benefits on sarcopenia severity are yet to be established.

Aerobic training, land- or water-based exercises in which muscle contractions are sustained for long periods, has long been known for its beneficial effects on cardiorespiratory health [18]. More recently, studies have examined the effects of aerobic-type exercises on sarcopenia with promising results [22,23]. However, the training protocols tested combined land-based aerobic exercise with other training modalities [22,23] and only studied men and people with specific conditions (e.g., COVID-19) [22]. Furthermore, no studies have examined the effects of water-based aerobic exercise or compared the effects of different exercise modalities on sarcopenia severity. Finally, the impact of exercise modalities on dynapenia assessed by IHG or STS has not yet been investigated.

To fill these gaps in knowledge, the present study was conducted to explore the association between different exercise modalities and sarcopenia severity in a large sample of Italian older adults living in the community.

## 2. Materials and Methods

The database of the Longevity Check-Up 7+ (Lookup 7+) project served as the data source for the current research. Sampling characteristics, methods, and other findings have been published previously [24,25,26,27,28,29]. Lookup 7+ began in June 2015 and has since been conducted in unconventional settings. People attending public places (e.g., exhibitions, malls, social events) and prevention campaigns launched by our department were invited to take part in the study. This sampling method was chosen to enroll relatively unselected participants, outside of conventional healthcare or research settings. The initiative has the objective of increasing awareness in the general population of major modifiable risk factors for chronic diseases and promote the adoption of healthy lifestyles. The manuscript was prepared in compliance with the STrengthening the Reporting of Observational studies in Epidemiology (STROBE) guidelines for observational studies [30].

### 2.1. Participants

From 1 June 2015 to 31 October 2021, we recruited 13,515 community-dwelling adults. Exclusion criteria were inability or unwillingness to provide written informed consent, self-reported pregnancy, and inability to complete the physical function tests requested by the protocol. For the present investigation, analyses were conducted using data from participants 65 years and older, with a body mass index (BMI) of at least 18.5 kg/m^2^, and no missing information for the variables of interest (*n* = 3289).

### 2.2. Data Collection

A structured interview was administered to collect information on lifestyle habits. Smoking status was categorized as current (has smoked 100 or cigarettes in lifetime and currently smokes) and non-current smoker. Healthy diet was operationalized as the consumption of three or more servings of fruit and/or vegetables per day [26,27]. Protein intake was estimated using a food frequency questionnaire (FFQ) [31]. Participants were questioned on their weekly intake of a standardized serving of 12 different foods, including meat, meat derivatives, fish, eggs, milk, cheese, yogurt, pasta, bread, rice, fruit and vegetables, and cereals. The serving size was according to the Italian standard portion reference [31]. For each food item, the protein intake per day was determined by multiplying its frequency of consumption by the amount of protein in a standard serving [31], then dividing this by seven (the days of a week). The mean protein intake was then calculated by summing all applicable items.

Standing height and body weight were measured using a standard stadiometer and an analog scale, respectively. The BMI was subsequently calculated as the ratio between body weight (in kg) and height squared (in m^2^).

### 2.3. Operationalization of Sarcopenia and Adherence to Exercise Modalities

Sarcopenia was identified according to the simultaneous presence of dynapenia and low appendicular muscle mass [1]. Muscle strength was assessed by IHG and STS testing. Participants underwent IHG testing while sitting comfortably on a chair with their shoulders neutral. The arm being assessed had the elbow flexed at 90° near the torso, and the hand neutral with thumbs up. A maximal contraction was performed over four seconds using a handheld hydraulic dynamometer (North Coast Hydraulic Hand Dynamometer; North Coast Medical, Inc., Morgan Hill, CA, USA) [32]. One familiarization trial was allowed before the actual testing. IHG was measured in both hands and the higher reading (reported in kg) was used for the analysis.

For the STS test, participants had to get up from a chair as quickly as they could while keeping their arms crossed at their chest. Timing was started when participants raised their buttocks off the chair and was halted when they were seated at the conclusion of the fifth stand [32].

Appendicular skeletal muscle mass (ASM) was estimated from the calf circumference of the dominant leg. The measure was taken at the largest girth between ankle and knee joints using an anthropometric tape while the participant was in a seated position. ASM was subsequently calculated according to the following formula [33]:

ASM = −10.427 + (calf circumference (cm) × 0.768) − (age × 0.029) + (sex (male = 1, female = 0)) × 7.523

The sex-specific cutoff points recommended by the EWGSOP2 were used to categorize muscle strength and ASM values [1]. Self-reported difficulty or inability to walk 400 m was the criterion used to define sarcopenia as severe.

Regular physical activity was defined as engaging in leisure-time physical activity at least twice a week for 30 min a session over the course of the previous year [31]. Participants who did not practice physical activity for 60 or more minutes at least twice weekly during the previous year were defined physically inactive (PI). Engagement in aerobic training was operationalized as the practice of running and/or swimming (RS) activities [34], whereas those who performed strength exercises with or without stretching (SS) were assigned to the resistance training group.

### 2.4. Statistical Analysis

The Shapiro–Wilk test was used to verify the normal distribution of variables. Continuous variables are expressed as mean ± standard deviation (SD), while categorical variables are reported as absolute numbers and percentages. Binary regression was conducted to test the association between IHG- and STS-based sarcopenia and training status. The final model was adjusted for age, sex, BMI, walking activity, smoking status, healthy diet, and daily protein intake. For all tests, the level of significance was set at 5% (*p* < 0.05). All *p*-values were determined by two-tailed tests. Confidence intervals (CIs) that included the number of 1 were not statistically significant. The SPSS software (version 23.0, SPSS Inc., Chicago, IL, USA) was used for all analyses.

## 3. Results

### 3.1. Participants Characteristics

The main characteristics of study participants according to sex and training groups are shown in Table 1. The mean age of participant was 72.7 ± 5.7 years and 55.1% were women. In women, the PI group had worse performance on the STS test in comparison to both RS and SS groups. Those who practiced RS were younger, stronger, and had lower BMI than their PI peers. No differences in any parameter were observed between exercise groups. A similar pattern of characteristics was observed in men, except for the lack of a significant difference in STS performance between SS and PI groups.

### 3.2. Associations between Physical Exercise and Sarcopenia Severity

The results of the binary regression for the association between training status and the presence of IHG- or STS-based sarcopenia in women and men are shown in Table 2 and Table 3, respectively. Neither RS or SS was associated with IHG-based sarcopenia in women. In contrast, a significant negative association was observed between RS and the presence of STS-based sarcopenia (odds ratio (OR) = 0.52, 95% CI = 0.26–0.95). In men, the training status was not significantly associated with STS-based sarcopenia. However, a significant negative association was found between RS and IHG-based severe sarcopenia (OR = 0.33, 95% CI 0.11–0.95).

## 4. Discussion

The main findings of the present study indicate that RS, but not SS, is negatively associated with the presence of sarcopenia in both men and women. Specifically, RS was negatively associated with STS-based sarcopenia in women and with IHG-based severe sarcopenia in men. These results suggest that muscle strength assessment tools and sex affect the relationship between physical exercise and the presence of sarcopenia.

Although the positive effects of exercise training on sarcopenia have consistently been observed [35], only a few investigations have considered sarcopenia severity as a study outcome. The lack of an association between SS and sarcopenia severity in our study was unexpected, given previous observations on a positive impact of strength training on muscle strength and physical performance in older adults with sarcopenia [16,19,20,21]. However, other investigations reported no or inconsistent improvements in muscle strength in older adults with sarcopenia engaged in resistance training programs [19,21]. Similarly, the effects of resistance training on muscle mass indexes are conflicting [16,20,21].

Altogether, the available evidence suggests that specific strength training protocols may be necessary to improve sarcopenia-related parameters and impact sarcopenia severity [36]. Substantial gains in neuromuscular function have been observed using exercise protocols that involve more sessions per week, with exercise repetitions performed next to failure at moderate-to-high intensity [37,38,39]. A recent meta-analysis reported that improvements in IHG strength are more likely to be observed in older adults with sarcopenia who attend strength training sessions for 40−60 min, at least three times per week for no less than 12 weeks [21].

On the other hand, we observed that adherence to aerobic exercise modalities was negatively associated with the presence of sarcopenia and severe sarcopenia. The effects of aerobic exercise alone on sarcopenia have been studied to a lesser extent than strength training. Indeed, exercise protocols that involve aerobic training for sarcopenic older adults are commonly combined with other exercise modalities [22,23].

Studies in individuals with chronic diseases or in non-sarcopenic older adults may help in the discussion of our results. For instance, Keogh et al. [40] and Alkatan et al. [41] found that continuous moderate-intensity aerobic training produced clinically significant improvements in physical function tests, including STS and IHG, in people with knee osteoarthritis. These findings were supported by Mangione et al. [42], who observed comparable improvements in gait speed and walking capacity, endurance fitness, and STS performance in older adults who followed a low- or moderate-intensity aerobic training program. Ballesta-Garcia et al. [43] reported that high-intensity interval training (HIIT) and moderate-intensity continuous aerobic training similarly improved STS and endurance performance in older women. However, increases in IHG strength were only observed after HIIT. Other investigations have also found that HITT induces larger gains in physical performance than continuous moderate-intensity exercise programs [40].

These observations suggest that the SS characteristics of our study participants were not adequate to yield improvements in sarcopenia. This idea is supported by the lack of significant associations also in the unadjusted analysis. However, because exercise variables were not controlled, we are unable to provide solid conclusions. Moreover, although the increased diffusion of exercise facilities [44,45] and online tutorials [46] stimulate people to exercise autonomously, supervised exercise may be necessary to efficiently improve neuromuscular function in older adults with sarcopenia [47].

In contrast, our data suggest that RS participants might have exercised under adequate conditions by performing aerobic sessions at moderate-to-high intensities. This is consistent with the lower presence of sarcopenia and severe sarcopenia in this participant subgroup. Studies with more specific designs (e.g., randomized clinical trials) are needed to confirm our interpretation.

The fact that the study outcomes were affected by the participant sex and the operationalization of dynapenia (i.e., IHG vs. STS) deserves attention due to possible clinical implications. Indeed, IHG and STS are not equivalent muscle strength proxies [10]. Several studies have noted that the associations between IHG strength with muscle strength of other segments and physical performance tests ranges from weak-to-moderate to high [4,5,6,7,8]. Moreover, IHG strength is a weak predictor of age-related decline in lower-limb muscle strength [6]. In contrast, STS performance is significantly associated with lower-limb muscle strength, muscle power, walking speed, and endurance [7,9,10,11,12]. Comparisons between these assessment tools suggest that STS is more strongly correlated with numerous physical performance tests [10]. These observations have led researchers to debate about the validity of IHG as a muscle strength measure [5,6,7,8,10]. Accordingly, IHG should be used in the initial clinical evaluation or as a complementary measure rather than as an indicator of neuromuscular function, especially for activities that do not require gripping [5,10].

The different physical attributes reflected by IHG and STS provide a possible explanation for our findings. RS involves cyclic movements that stimulate muscle groups involved in STS performance [48], mainly hip, knee, and ankle extensors and torso stabilizers [49,50,51]. RS activities also ameliorate intra- and inter-muscular coordination of upper limbs, which provide stability during STS testing [48], as well as hip flexion and rotation during the acceleration phase [50,51]. None of these tasks involve gripping, which explains why RS had negligible effects on IHG strength.

Swimming involves numerous upper limb tasks performed against water resistance, including specific movements of wrist and hands to create propulsion during freestyle, butterfly, backstroke, and breaststroke modalities [51]. In addition, the use of equipment to improve swimming technique and performance (e.g., kickboard) requires gripping. It might be speculated that specific swimming exercises could have improved IHG strength, whereas endurance swimming may have positively impacted the STS performance. In fact, such activities might be sufficient to avoid severe sarcopenia in men. However, more specific tasks might be required for sarcopenia. On the other hand, RS activities at adequate conditions might require the supervision of an exercise trainer to avoid sarcopenia [47]. Notably, these premises are only hypothetical.

A complementary explanation to our findings is based on the different patterns of occupational activities performed by men and women [52]. Domestic activities often represent a large proportion of total physical activity in women, whereas men are frequently more involved in recreational and occupational tasks [52,53]. In addition, when men and women spend similar periods performing domestic activities, women do more heavy work than men [54]. These observations might explain why STS-based sarcopenia was negatively associated with RS in women, but not in men. Indeed, household tasks encompass many lower limb-based activities, including cleaning, STS, and stair climbing [52].

The present study is not free of limitations. First, physical exercise variables were not controlled. Second, our sample was exclusively composed of relatively young community-dwelling Caucasian older adults, and extrapolation to other age groups or ethnicities should be considered with caution. Third, combining IHG and STS testing might offer a more comprehensive appraisal of whole-body muscle strength than any of them alone [10]. Fourth, ASM was estimated using an equation based on calf circumference. Fifth, participant-reported information was used to determine sarcopenia severity. This aspect deserves concern, given that self-report and performance-based physical performance measures capture different neuromuscular aspects [55]. Sixth, we cannot exclude that our results could be more associated with occupational activities than physical activity levels. Seventh, participants were evaluated while they were attending an event, which might have impacted their IHG and STS performance. Finally, the cross-sectional design of the study does not allow any inference to be drawn on the time course of changes in the variables considered or on cause–effect relationships.

## 5. Conclusions

Findings of the present study indicate that RS is negatively associated with the presence of sarcopenia in a large sample of relatively unselected Italian older adults. Specifically, RS was negatively associated with STS-based sarcopenia in women and with IHG-based severe sarcopenia in men. These results suggest that muscle strength assessment tools and sex affect the relationship between physical exercise and the presence of sarcopenia.

## Figures and Tables

**Table 1:** Main characteristics of study participants according to sex and training groups (*n* = 3289).

	Women (*n* = 1814)			Men (*n* = 1475)		
	PI (*n* = 1588)	RS (*n* = 198)	SS (*n* = 28)	PI (*n* = 1168)	RS (*n* = 285)	SS (*n* = 22)
Age, years	72.6 ± 5.6	71.3 ± 4.8 ^b^	71.5 ± 6.0	73.4 ± 5.9	71.7 ± 5.3 ^c^	72.5 ± 5.4
BMI, kg/m^2^	26.2 ± 4.5	25.3 ± 4.0 ^a^	24.5 ± 3.8	27.0 ± 3.6	25.9 ± 3.2 ^a^	26.8 ± 3.9
STS test, s	9.7 ± 3.7	7.9 ± 1.8 ^c^	7.9 ± 1.8 ^b^	9.1 ± 2.7	8.2 ± 2.0 ^c^	7.8 ± 1.8
IHG, kg	20.2 ± 5.3	21.1 ± 5.1 ^a^	22.9 ± 8.4	34.4 ± 7.9	36.5 ± 7.8 ^c^	35.2 ± 9.1
ASM, kg/m^2^	5.4 ± 1.1	5.4 ± 0.9	5.3 ± 0.8	7.7 ± 0.9	7.5 ± 0.8	7.3 ± 1.1
Protein intake, g/kg of BW/day	0.62 ± 0.20	0.62 ± 0.18	0.64 ± 0.17	0.52 ± 0.15	0.52 ± 0.21	0.52 ± 0.16

Abbreviations: ASM, appendicular skeletal muscle mass; BMI, body mass index; BW, body weight; IHG, isometric handgrip strength; PI, physically inactive; STS, sit-to-stand; RS, running and/or swimming; SS, strength training with or without stretching exercises. ^a^ *p* < 0.05 vs. PI; ^b^ *p* < 0.01 vs. PI; ^c^ *p* < 0.001 vs. PI.

**Table 2:** Association between training status and sarcopenia in older women (*n* = 1814).

	Unadjusted OR (95% CI)	*p*-Value	Adjusted OR (95% CI) *	*p*-Value		Unadjusted OR (95% CI)	*p*-Value	Adjusted OR (95% CI) *	*p*-Value
IHG-based sarcopenia					STS-based sarcopenia				
PI	1.00 (Reference)	—	1.00 (Reference)	—	PI	1.00 (Reference)	—	1.00 (Reference)	—
RS	1.29 (0.78, 2.13)	0.321	1.30 (0.70, 2.44)	0.397	RS	0.60 (0.16, 2.25)	0.454	0.50 (0.26, 0.95)	0.036
PI	1.00 (Reference)	—	1.00 (Reference)	—	PI	1.00 (Reference)	—	1.00 (Reference)	—
SS	3.10 (0.86, 11.14)	0.082	2.12 (0.54, 8.32)	0.279	SS	0.57 (0.32, 1.00)	0.050	0.57 (0.14, 2.32)	0.435
IHG-based severe sarcopenia					STS-based severe sarcopenia				
PI	1.00 (Reference)	—	1.00 (Reference)	—	PI	1.00 (Reference)	—	1.00 (Reference)	—
RS	0.67 (0.31, 1.41)	0.296	0.72 (0.29, 1.80)	0.493	RS	0.65 (0.13, 3.05)	0.585	0.69 (0.13, 3.54)	0.661
PI	1.00 (Reference)	—	1.00 (Reference)	—	PI	1.00 (Reference)	—	1.00 (Reference)	—
SS	0.00 (0.00)	0.999	0.00 (0.00)	0.999	SS	0.41 (0.19, 0.88)	0.023	0.47 (0.19, 1.12)	0.090

Abbreviations: CI, confidence interval; IHG, isometric handgrip strength; OR, odds ratio; PI, physically inactive; RS, running and/or swimming; SS, strength training with or without stretching exercises; STS, sit-to-stand. * Models were adjusted for age, body mass index, smoking status, healthy diet, daily protein intake, and walking activity.

**Table 3:** Association between training status and sarcopenia in older men (*n* = 1475).

	Unadjusted OR (95% CI)	*p*-Value	Adjusted OR (95% CI) *	*p*-Value		Unadjusted OR (95% CI)	*p*-Value	Adjusted OR (95% CI) *	*p*-Value
IHG-based sarcopenia					STS-based sarcopenia				
PI	1.00 (Reference)	—	1.00 (Reference)	—	PI	1.00 (Reference)	—	1.00 (Reference)	—
RS	1.10 (0.75, 1.61)	0.600	0.79 (0.49, 1.29)	0.357	RS	0.60 (0.16, 2.25)	0.345	0.52 (0.26, 0.95)	0.008
PI	1.00 (Reference)	—	1.00 (Reference)	—	PI	1.00 (Reference)	—	1.00 (Reference)	—
SS	0.69 (0.17, 2.71)	0.597	0.27 (0.04, 1.76)	0.175	SS	0.50 (0.10, 2.50)	0.400	0.57 (0.14, 2.32)	0.582
IHG-based severe sarcopenia					STS-based severe sarcopenia				
PI	1.00 (Reference)	—	1.00 (Reference)	—	PI	1.00 (Reference)	—	1.00 (Reference)	—
RS	0.52 (0.13, 0.76)	0.011	0.33 (0.11, 0.95)	0.041	RS	0.65 (0.13, 3.05)	0.523	0.69 (0.13, 3.54)	0.533
PI	1.00 (Reference)	—	1.00 (Reference)	—	PI	1.00 (Reference)	—	1.00 (Reference)	—
SS	0.68 (0.08, 5.63)	0.721	1.34 (0.12, 14.0)	0.806	SS	0.000 (0.000)	0.999	0.000 (0.000)	0.999

Abbreviations: CI, confidence interval; IHG, isometric handgrip strength; OR, odds ratio; PI, physically inactive; RS, running and/or swimming; SS, strength training with or without stretching exercises; STS, sit-to-stand. * Models were adjusted for age, body mass index, smoking status, healthy diet, daily protein intake, and walking activity.

## Data Availability

Data are available upon reasonable request from the corresponding authors.
